# Female With Severe Abdominal Pain

**DOI:** 10.1016/j.acepjo.2025.100075

**Published:** 2025-03-01

**Authors:** Dana Mathew, Kimberly Ciccone

**Affiliations:** 1 Burrell College of Osteopathic Medicine Melbourne, Satellite Beach, Florida, USA; 2 University of North Florida, Jacksonville, Florida, USA

**Keywords:** vaginal cuff dehiscence, hysterectomy, laparoscopic hysterectomy

## 1 Patient Presentation

A 52-year-old female presented to the emergency department with a chief complaint of severe abdominal pain. The patient reported sudden onset of nausea, vomiting, vaginal pain, and bleeding during intercourse. She had a distant history of cervical cancer. Physical examination was limited secondary to pain but remarkable for diffuse tenderness about the abdomen with guarding. There appeared to be a bowel protruding into the vagina.

## 2 Diagnosis*:* Vaginal Cuff Dehiscence

**Figure 1:**
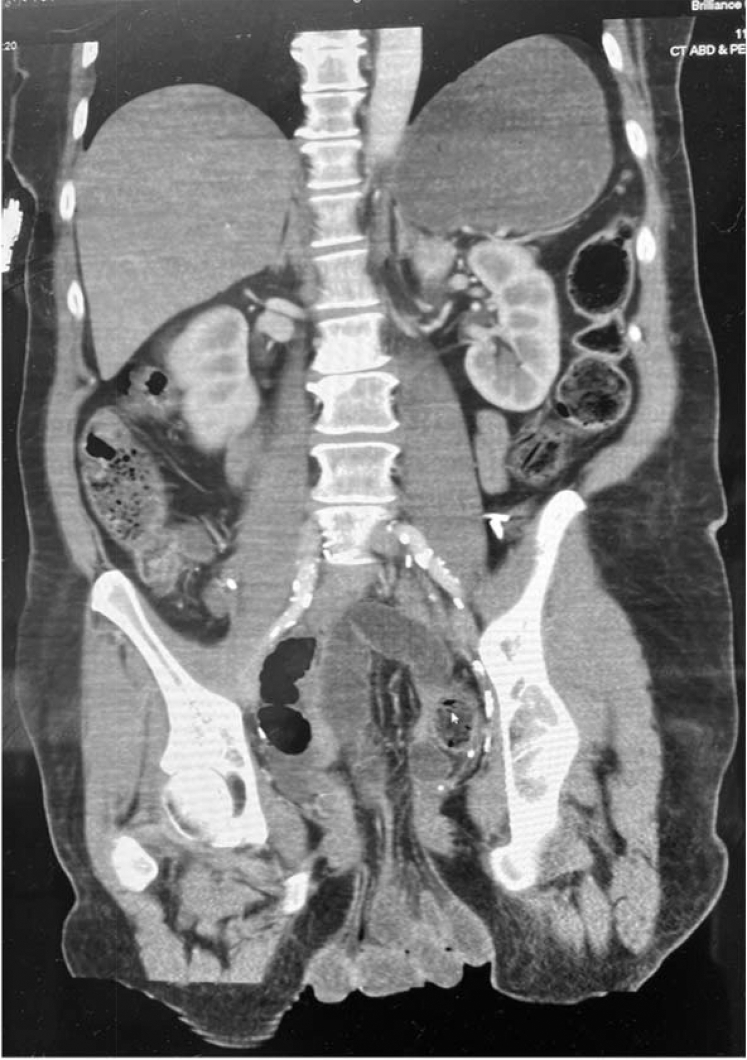
Computed tomography (CT) of the abdomen and pelvis of bowel evisceration secondary to vaginal cuff dehiscence, coronal view.

The computed tomography (CT) confirmed vaginal cuff dehiscence (VCD) with small bowel evisceration (Fig 1). The patient was immediately transferred to gynecology oncology for emergent surgery. The patient underwent a successful reduction and repair with discharge later that day.

Hysterectomies are one of the most common surgeries in the United States.^1^ The occurrence of VCD with evisceration is rare, ranging from 0.032% to 1.2%.^2^ Increased risk is associated with multiple factors, including malignancy, comorbidities affecting wound healing, surgical techniques, coitus, and increased intraabdominal pressure.^3^ Our patient presented with a history of cervical cancer, the fourth most common cancer impacting women worldwide.^4^ Over the last decade, laparoscopic radical hysterectomy evolved as the primary treatment method for early-stage cervical cancer.^5^^,^^6^ Total laparoscopic hysterectomies have an increased prevalence of VCD risk in comparison with other surgical modalities.^1^^,^^7^^,^^8^ Evisceration of the bowel through the vaginal canal can lead to severe sequelae, including peritonitis, sepsis, bowel injury, and necrosis. VCD with evisceration is a surgical emergency. Early recognition and rapid surgical management are essential to avoid serious complications.

## Funding and Support

By *JACEP Open* policy, all authors are required to disclose any and all commercial, financial, and other relationships in any way related to the subject of this article as per ICMJE conflict of interest guidelines (see www.icmje.org). The authors have stated that no such relationships exist.

## Conflict of Interest

All authors have affirmed they have no conflicts of interest to declare.
